# Effect of Acute and Prolonged Inflammation on the Gene Expression of Proinflammatory Cytokines and Their Receptors in the Anterior Pituitary Gland of Ewes

**DOI:** 10.3390/ijms21186939

**Published:** 2020-09-21

**Authors:** Karolina Wojtulewicz, Agata Krawczyńska, Dorota Tomaszewska-Zaremba, Maciej Wójcik, Andrzej P. Herman

**Affiliations:** The Kielanowski Institute of Animal Physiology and Nutrition, Polish Academy of Sciences, Instytucka 3, 03-105 Jabłonna, Poland; a.krawczynska@ifzz.pl (A.K.); d.tomaszewska@ifzz.pl (D.T.-Z.); m.wojcik@ifzz.pl (M.W.); a.herman@ifzz.pl (A.P.H.)

**Keywords:** inflammation, cytokine, anterior pituitary, gonadotropins

## Abstract

An acute and prolonged inflammation inhibits the reproduction process by the disruption of the neurohormonal activity of the hypothalamic-pituitary-gonadal axis. It is thought that these changes may be caused by proinflammatory cytokines, i.e., interleukin (IL) -1β, IL-6 and tumor necrosis factor (TNF) α. The aim of this study was to determine the effect of an acute and prolonged inflammation on the expression of genes encoding cytokine and their receptors, gonadotropin releasing hormone receptor (*GnRHR*), beta subunits of luteinizing hormone (*LHβ*) and follicle-stimulating (*FSHβ*) in the anterior pituitary (AP). Moreover, the circulating concentration of LH and FSH was also assayed. Two experiments were carried out on adult ewes which were divided into two control groups and treated with lipopolysaccharide (LPS; 400 ng / kg). Acute inflammation was caused by a single injection of LPS into the external jugular vein, while the chronic inflammation was induced by seven times LPS injection (one a day). In both experiments, animals were euthanized 3h after the last LPS / NaCl injection and the blood samples collected 15 min before euthanasia. An acute inflammation stimulates the expression of the *IL-1β*, *IL-6* and *TNFα* genes and their receptors in the AP of sheep. Prolonged inflammation increased *TNFα* gene expression and both types of *TNFα* and *IL-6* receptors. Both an acute and prolonged inflammation inhibited LHβ gene expression in the AP and reduced LH level in blood. A sevenfold LPS injection raises FSH concentration. The gene expression of *GnRHR* was reduced in the ovine AP only after a single injection of endotoxin. Our results suggest that there are important differences in the way how an acute and prolonged inflammation influence proinflammatory cytokines and their receptors gene expression in the AP of anestrous ewes, which could be reflected by differences in the AP secretory activity during these states.

## 1. Introduction

An acute and prolonged inflammation affects endocrine system functioning in numerous animal species, including sheep [1,2]. Circulating inflammatory mediators, such as interleukin (IL) -1β, IL-6 and tumor necrosis factor (TNFα), play an important role in the induction of endocrine disorders during inflammation. It was determined that these pro-inflammatory mediators might affect the reproductive process via the alteration of the hypothalamic-pituitary-gonadal axis activity [3,4,5]. This action of pro-inflammatory cytokines may occur both at the hypothalamic [6] and pituitary level [7]. However, the recent studies conducted on sheep suggest that an acute and prolonged inflammation may influence gonadotropins secretion in different ways. A single administration of endotoxin suppresses circulating concentration of luteinizing hormone (LH) in a number of species, including rats, sheep, cattle, and non-human primates [1,8,9]. There is no effect of an LPS injection on FSH plasma concentration in rats and sheep, while in sheep the gene expression of follicle-stimulating hormone (*FSH*) β was increased [6,8]. On the other hand, the study with a prolonged administration of endotoxin showed that the plasma concentration of FSH grew up from second day in LPS-treated ewes, while the LH release stayed decreased [10]. On the other hand, a prolonged inflammation induced by LPS injection in primates (Rhesus Monkey) caused an increase in circulating concentration of both FSH and LH [11].

It is well proven that inflammation modulates the reproduction process affecting the GnRH secretion at the level of hypothalamus. It was described that an LPS-induced acute inflammation inhibited the LH release via suppression of the pulsatile secretion of GnRH in the hypothalamus of sheep [1]. The results of our previous study suggest that the reduction of GnRH secretion occurring during an immune/inflammatory challenge might result from the inhibition of this decapeptide synthesis at the level of its gene transcription in the preoptic area by centrally acting IL-1β [12]. However, a recent ex vivo study showed that proinflammatory cytokines may also influence LH secretion directly at the level of pituitary. It was found that IL-1β reduced GnRH-stimulated the LH secretion from the anterior pituitary (AP) explants [5], while IL-6 stimulated the LH release from pituitary explants [13]. The pro-inflammatory cytokines may act on the pituitary cells due to the existence of their corresponding receptors in cell membranes [7,14,15]. Our previous study showed that mRNA encoding proinflammatory cytokines could be transcribed directly in the pituitary [14]; therefore, the local expression of these mediators as well as their corresponding receptors could have a profound influence on the secretory activity of this gland especially during inflammatory states.

The aim of this study was to determine the influence of an acute and prolonged inflammation on the expression of genes encoding pro-inflammatory cytokines such as IL-1β, IL-6, TNFα and their corresponding receptors, LH, FSH and GnRH receptor in the AP and the circulating concentration of LH and FSH. The study was performed on blackhead ewes model because, in contrast to rodents, the sensitivity of sheep to the action of endotoxins is similar to humans. Therefore these animals are increasingly used in the studies on many diseases including sepsis, asthma pathogenesis, vaccine development and the optimization of drug delivery and surgical techniques [16,17].

## 2. Results

### 2.1. Influence of Acute and Prolonged Inflammation on IL1B, IL1R1 and IL1R2 Gene Expression

**Figure 1:**
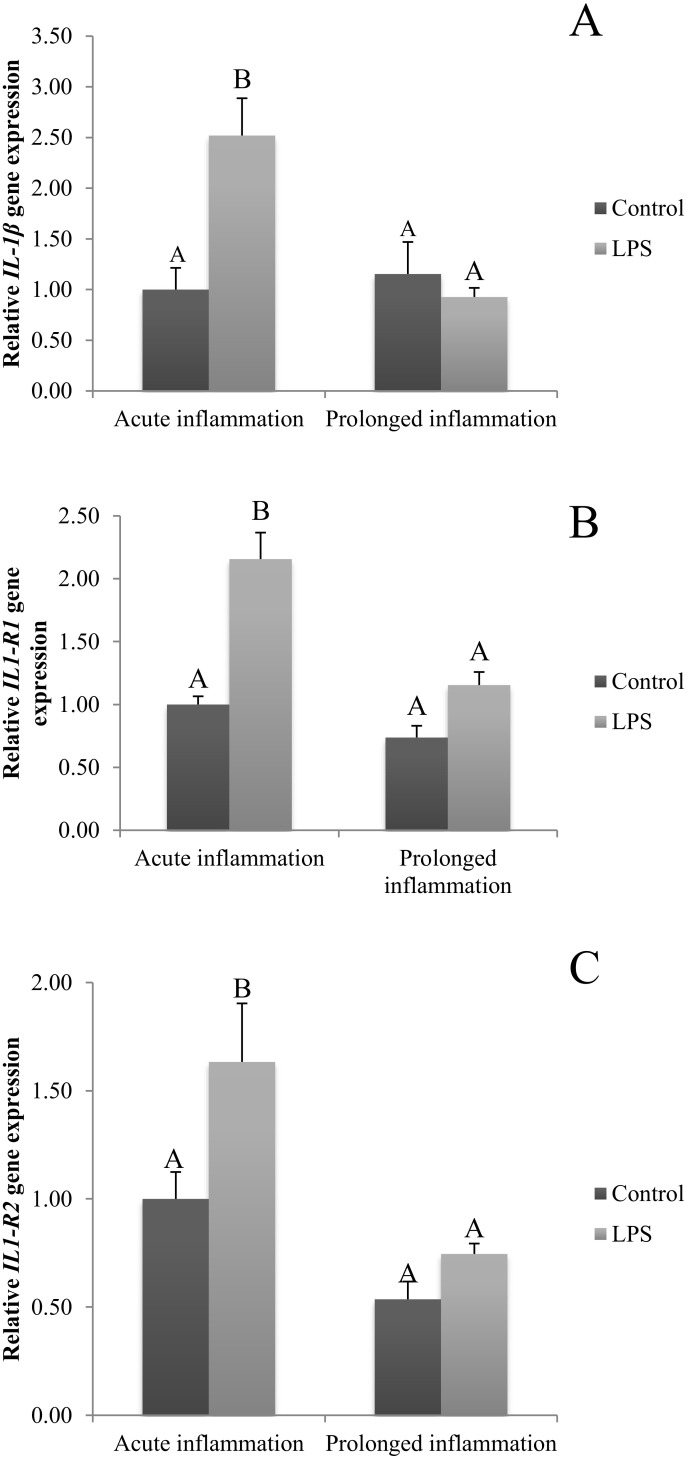
The effects of an acute and prolonged inflammation on the relative IL1β (**A**) and their receptors IL1R1 (**B**) and IL1R2 (**C**) mRNA levels in the AP normalized to the average value of three reference genes expression: actin beta (ACTB), glyceraldehyde 3-phosphate dehydrogenase (GAPDH), peptidylprolyl isomerase C (PPIC). The data are presented as a mean value ± S.E.M.; Significantly according to the multiple analysis of variance followed by a Tuckey’s post-hoc test (*p* < 0.05). ABC-bars with different letters vary.

The expression of IL1β was stimulated (*p* < 0.05) by a single injection of LPS (Figure 1A). Moreover, the expression of mRNA for both types of IL-1 receptors (IL1R1 and IL1R2) is increased during an acute inflammation (Figure 1B,C). On the other side, a sevenfold injection of endotoxin caused no effect on IL1β mRNA expression (Figure 1A). There is also no difference in IL1R1 and IL1R2 gene expression during prolonged stress compared to the control group (Figure 1B,C).

### 2.2. Influence of Acute and Prolonged Inflammation on IL-6, IL-6R and IL-6ST Gene Expression

**Figure 2:**
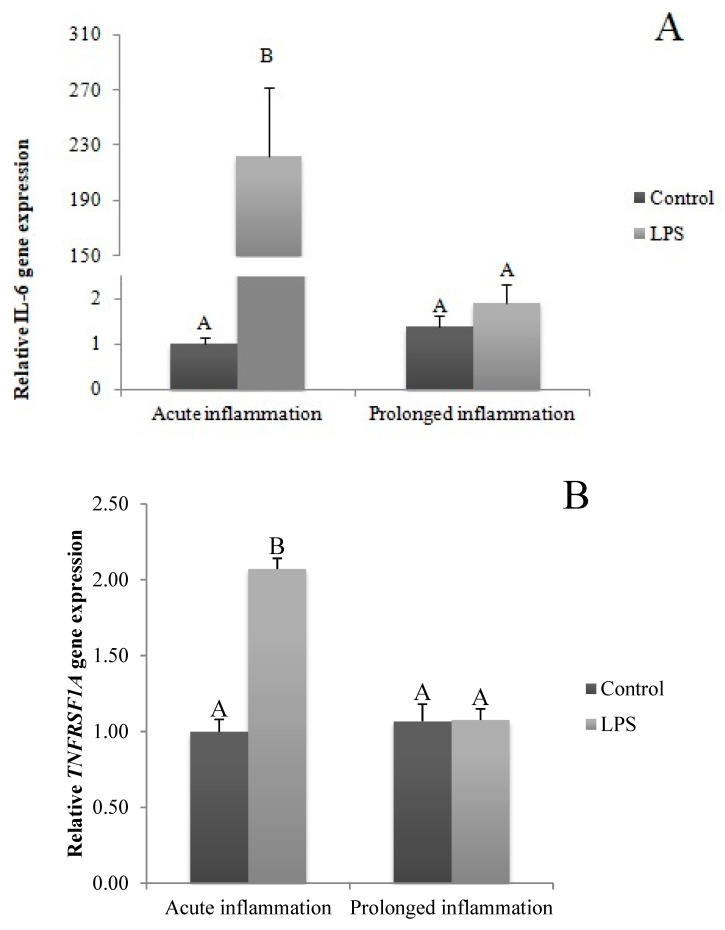

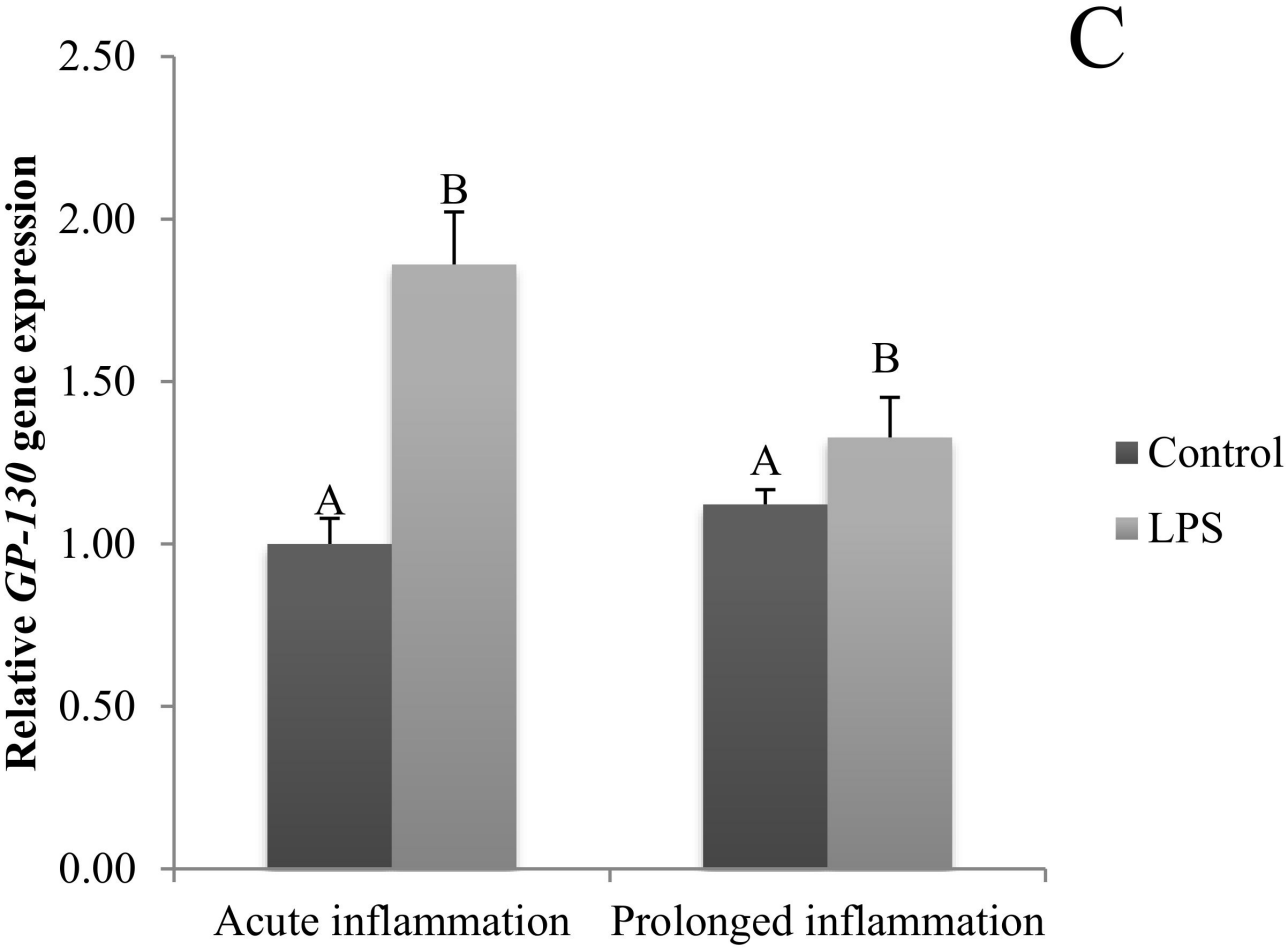
The effects of an acute and prolonged inflammation on the relative IL6 (**A**) and their receptors IL6R (**B**) and IL6ST (**C**) mRNA levels in the AP normalized to the average value of three reference genes expression: actin beta (ACTB), glyceraldehyde 3-phosphate dehydrogenase (GAPDH), peptidylprolyl isomerase C (PPIC). The data are presented as a mean value ± S.E.M.; Significantly according to the multiple analysis of variance followed by a Tuckey’s post-hoc test (*p* < 0.05). ABC-bars with different letters vary.

A single LPS injection increased (*p* < 0.05) IL6 gene expression (Figure 2A). The level of IL6R (Figure 2B) and IL6ST (Figure 2C) mRNA expression was higher (*p* < 0.05) during an acute inflammation compared to the control group. The prolonged inflammation has no effect on IL6 mRNA expression (Figure 2A). However, a sevenfold LPS injection enhanced (*p* < 0.05) IL6R (Figure 2B) and IL6ST (Figure 2C) gene expression in comparison to the control group.

### 2.3. Influence of Acute and Prolonged Inflammation on TNF, TNFRSF1A and TNFRSF1A Gene Expression

**Figure 3:**
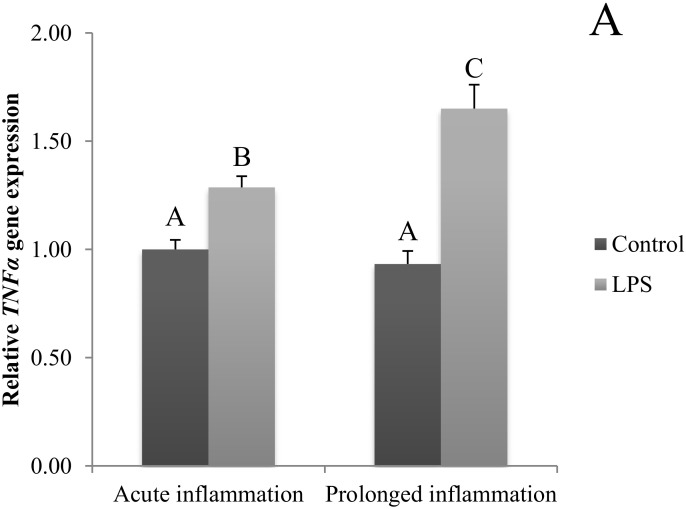

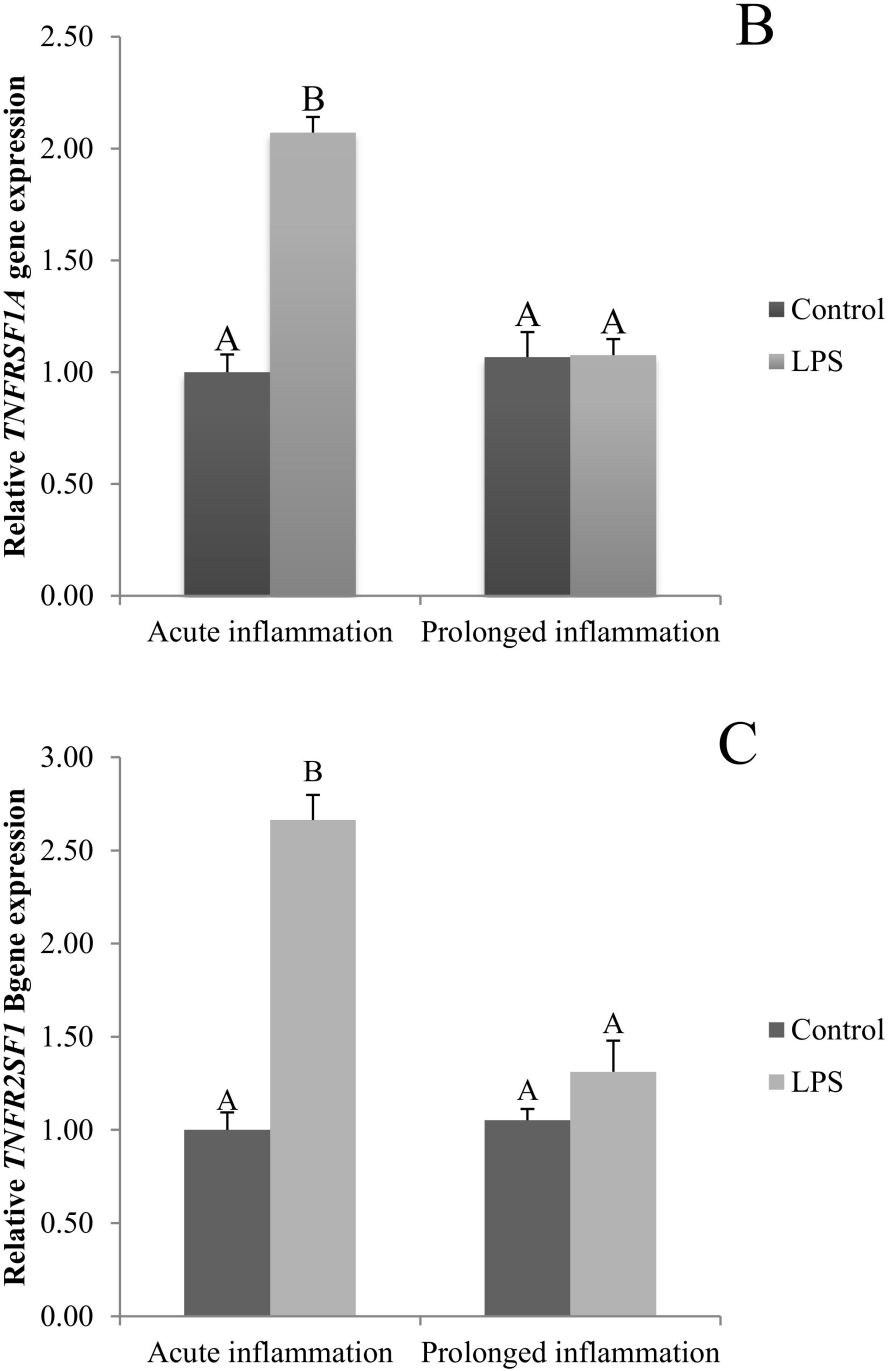
The effects of an acute and prolonged inflammation on the relative TNFα (**A**) and their receptors TNFRSF1A (**B**) and TNFRSF1B (**C**) mRNA levels in the AP normalized to the average value of three reference genes expression: actin beta (ACTB), glyceraldehyde 3-phosphate dehydrogenase (GAPDH), peptidylprolyl isomerase C (PPIC). The data are presented as a mean value ± S.E.M.; Significantly according to the multiple analysis of variance followed by a Tuckey’s post-hoc test (*p* < 0.05). ABC-bars with different letters vary.

The TNFα gene expression was stimulated (*p* < 0.05) during an acute inflammation (Figure 3A). Both TNF receptors (TNFRSF1A and TNFRSF1B) gene expression was enhanced (*p* < 0.05) also by a single endotoxin injection (Figure 3B,C). Sevenfold administration of an inflammatory factor enhanced (*p* < 0.05) TNFα gene expression (Figure 3A). Furthermore, this expression is stronger (*p* < 0.05) even in comparison to TNFα gene expression in the acute inflammation group. There is no effect of prolonged inflammation on TNFRSF1A (Figure 3B) and TNFRSF1B (Figure 3C) gene expression.

### 2.4. Influence of Acute and Prolonged Inflammation on LHβ, FSHβ and GnRHR Gene Expression

**Figure 4:**
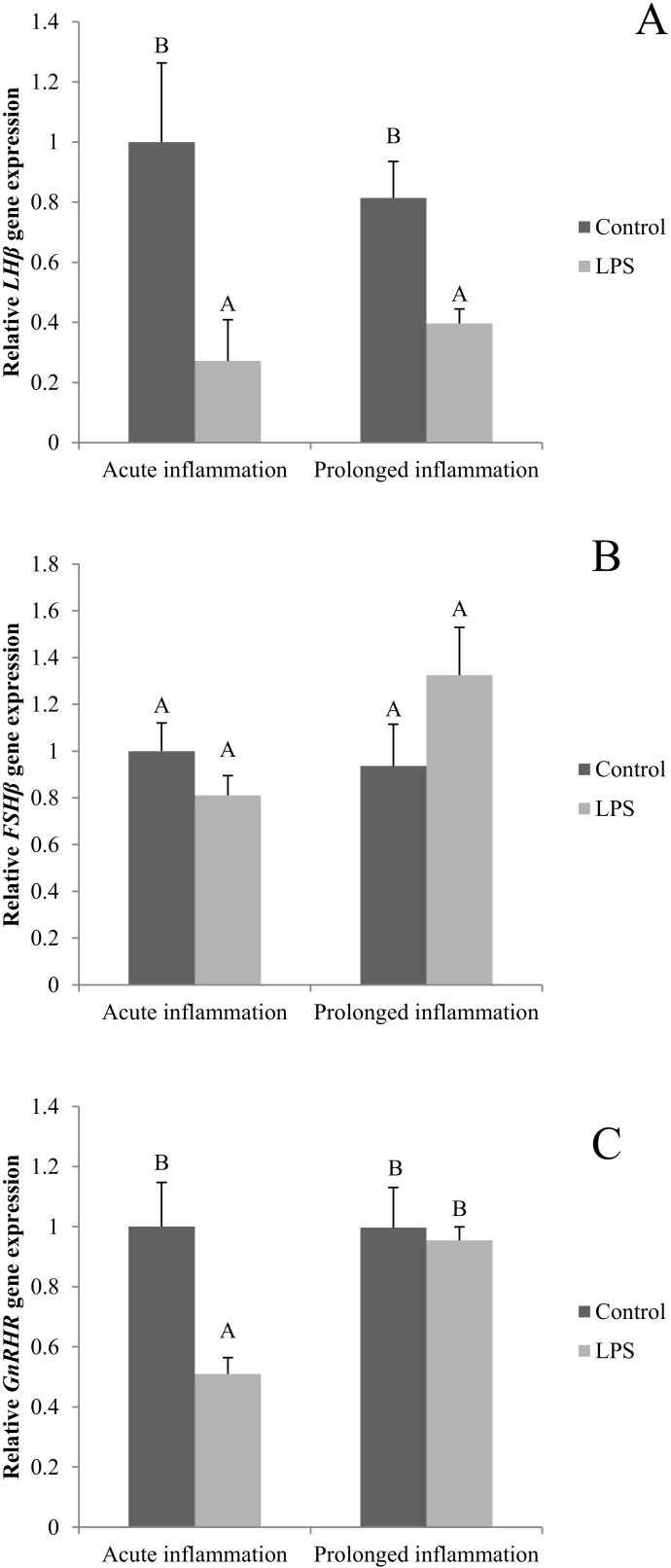
The effects of an acute and prolonged inflammation on the relative *LHβ* (**A**) *FSHβ* (**B**) and *GnRHR* (**C**) mRNA levels in the AP normalized to the average value of three reference genes expression: actin beta (*ACTB)*, glyceraldehyde 3-phosphate dehydrogenase (*GAPDH*), peptidylprolyl isomerase C (*PPIC*). The data are presented as a mean value ± S.E.M.; Significantly according to the multiple analysis of variance followed by a Tuckey’s post-hoc test (*p* < 0.05). AB-bars with different letters vary.

A single LPS injection inhibited (*p* < 0.05) both *LHβ* (Figure 4A) and *GnRHR* (Figure 4C). No effect of an acute inflammation was observed on *FSHβ* gene expression (Figure 4B). A sevenfold LSP administration resulted in the reduction (*p* < 0.05) of *LHβ* gene expression level. The level of *LHβ* gene expression was similar during the acute and prolonged inflammation. There was no effect of the prolonged inflammation of FSHβ (Figure 4B) and *GnRHR* (Figure 4C) gene expression.

### 2.5. Influence of Acute and Prolonged Inflammation on LH and FSH Concentration in Blood

**Figure 5:**
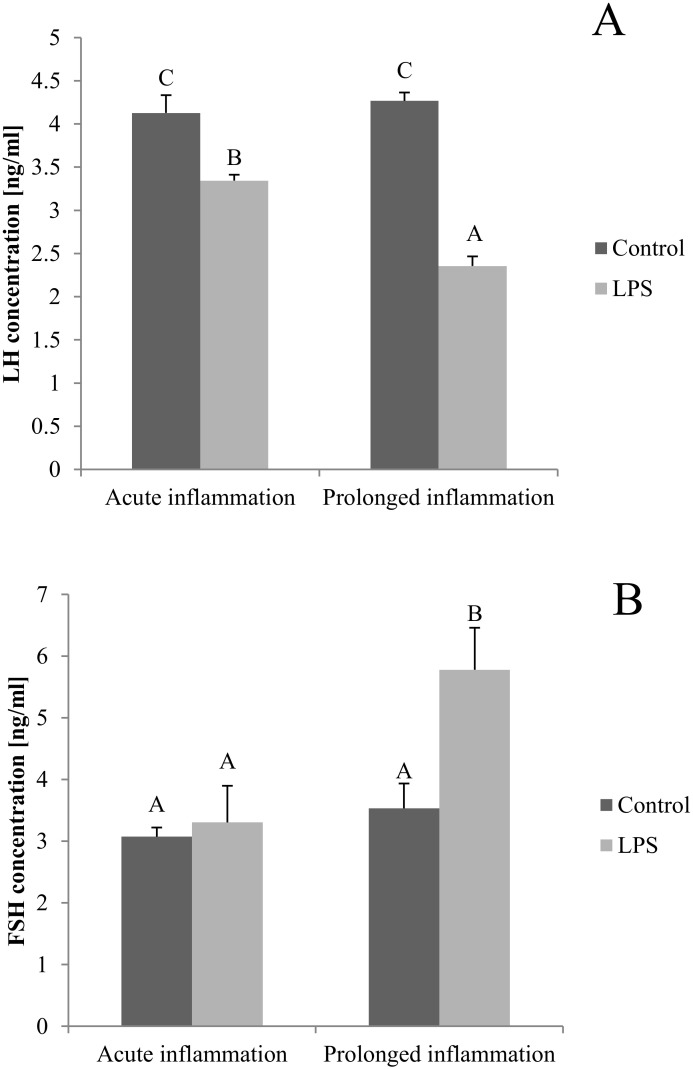
The effects of an acute and prolonged inflammation on the LH (**A**) and FSH (**B**) centration in blood. The data are presented as a mean value ± S.E.M.; Significantly according to the multiple analysis of variance followed by a Tuckey’s post-hoc test (*p* < 0.05). ABC-bars with different letters vary.

The blood concentration of LH was lower (*p* < 0.05) after both, a single and a sevenfold LPS injection. Additionally, the inhibitory effect of LPS was stronger (*p* < 0.05) during the prolonged inflammation (Figure 5A). There is no effect of single LPS treatment, while the sevenfold LPS injection raises (*p* < 0.05) the FSH concentration level (Figure 5B).

## 3. Discussion

Our study showed that mRNA encoding proinflammatory cytokines is transcribed in the significant amount in the ovine AP, both in the physiological state as well as during inflammation. This finding supports the results of our previous study showing the existence of nocturnal pro-inflammatory cytokine and their receptors gene expression in ovine Pars Tuberalis (PT) cells [14]. Moreover, the expression of interleukin in the pituitary had been previously reported in the study on the rat and mouse [15,18,19]. Studies on rats and mice AP showed that IL-1β, IL-6 and TNFα are secreted mainly by folliculo-stellate (FS) cells [20], moreover the in vitro study on this cells showed that folliculo-stellate cells are rich source of IL-6 and this cytokine is produced without an intentional stimulation [21]. On the other hand, the synthesis of IL1 mRNA was detected not only in FS cells but also in pituicytes and thyrotropes of rat [22]. Meanwhile, the study on human showed the expression of mRNA encoding proinflammatory cytokines in the pituitary tumor cells [7]. The expression of proinflammatory cytokines can be modulated by numerous factors, including cytokines themselves or LPS [23].

However, the majority of the studies analyzed only the effect of an acute exposure to LPS action on the pituitary cells. In 1993, Takao et. al., found that single LPS treatment enhanced *IL-1β* synthesis in mouse pituitary, spleen and testis, while the concentration of IL-1β in hypothalamus and hippocampus did not change after one LPS injection. In the second part of the experiment, the scientists determined if the LPS-induced increases in IL-1β concentration altered IL-1 receptors. It turned out that a double injection caused a significant decrease in [125I] IL-1 alpha binding using quantitative autoradiography in pituitary [15]. The study on mice also showed that the injection of LPS enhanced both: the serum level of *IL-1β, IL-6* and *TNFα* and the gene expression of these cytokines in the hypothalamus [24], pituitary and peripheral tissue [18]. Moreover, it was found that an intratracheal injection of LPS also upregulates *IL-1β* and *TNFα* expression in rat lung [25], IL-6 in rat [26] and mouse [27] liver.

Our study showed that there are significant differences between the effect of an acute and prolonged LPS-treatment on the gene expression of proinflammatory cytokines and their receptors in the ovine anterior pituitary. It was found that a single LPS injection increased the transcription of all examined cytokines, which is similar to other experiments conducted on sheep [28]. In contrast to acute inflammation when the gene expression of all examined cytokines was increased (the transcription of *IL-6* and *IL-1β* was increased more than *TNFα*), prolonged administration of bacterial endotoxin did not influence *IL-1β* and *IL-6* gene expression in the anterior pituitary. On the other hand, the prolonged LPS treatment significantly increased the gene expression of *TNFα* in the AP whereas the stimulatory effect of the acute LPS administration was on this gene expression was weaker. It is worth mentioning that the lack of a stimulatory effect of a prolonged inflammation on the gene expression of *IL-1β* and *IL*-6 in the pituitary differs from the previous observation made in other brain structure—hypothalamus. These experiment showed that a sevenfold injection of LPS increased the synthesis of *IL-1β, IL-6* and *TNFα* in ovine hypothalamus [29]. Moreover, in the hypothalamus the LPS-dependent induction of *IL-6* synthesis was stronger than in the case of *IL-1β* and *TNFα*. These observations wew also supported by the study on mice analyzing the pattern of cytokine synthesis in the brain after a repetitive LPS injection. This study shows that the brain and serum cytokine and chemokine profiles differed in mice receiving a single LPS injection compared to multiple LPS injections. In a mouse brain, three injections of LPS elevated *IL-1α, IL-6*, and *TNFα* compared to controls and the values for a single LPS injection [30].

The obtained results suggest that a different response of the endocrine system during an acute and prolonged inflammation at least partially may result from the differences in the local synthesis of proinflammatory cytokines in the pituitary gland. Whereas during an acute inflammation the secretory activity of the pituitary may be dependent upon the local synthesis and action of all examined proinflammatory mediators, during a prolonged inflammation this modulatory action seems to be primarily mediated by TNFα. It is well known that an acute and chronic inflammation is accompanied by an increased production of TNFα [31,32]. Experiments with the knock-in mice with a deletion that results in TNF overproduction, showed the development of chronic inflammatory arthritis and inflammatory bowel disease [33]. It is worth mentioning that the blockade of TNFα synthesis is considered to be an effective way to therapy of many disorders which are accompanied by the chronic inflammation [32,34]. Based on our findings it seems to be reasonable that a therapy focused on inhibiting TNFα synthesis could have some beneficial effect on the endocrine disorders which commonly accompany prolonged inflammatory states, such as reproduction disorders.

*IL-1β* gene expression is commonly induced in the brain after LPS treatment. The regions of blood–cerebrospinal fluid barrier had an early and sustained induction of *IL-1β* gene expression, while regions of the brain lacking BBB are characterized by only an early increase of this gene expression. In the hypothalamus, a remarkable increase of *IL-1β* mRNA 6 h after an LPS injection was observed, while in the pituitary gland high levels of the induction of IL-1β mRNA in the posterior pituitary, with little *IL-1* receptor agonist (ra), *IL-10*, or *IL-13* gene expression was found. In contrast, in the AP it was found that a systemic inflammation induced *IL-1ra* mRNA in the levels that were much higher than those for *IL-1β* mRNA [35].

Specific receptors are needed for cytokines to properly affect cell functioning. In mice and human, two distinct types of IL-1 receptors (IL-1R) have been characterized. Most of the IL-1 signal is transmitted through IL-1R and IL-1R2 and may act as a suppressor of IL-1β biological activities by competing in binding with IL-1R1 on the cell surface [36]. IL-6 initially binds to the membrane bound, or non-signal-transducing IL-6R. The complex of IL-6 and IL-6R associates with the signal transducing membrane protein gp130, promoting its dimerization and the initiation of intracellular signaling [37,38]. Two distinct receptors are required to exert TNF bioactivity. It is considered that TNFR1 promotes inflammation and tissue degeneration, while TNFR2 mediates local homeostatic effects, such as cell endurance and tissue regeneration [31,39]. All those receptors are present in the pituitary gland [40]. Our earlier study showed that the transcripts encoding proinflammatory cytokines and their receptors were expressed in the PT. Moreover, the LPS administration during a long night caused a higher expression of *IL6, TNF, IL6ST, TNFRSF1A* and *TNFRSF1B* genes compared to that in the tissues collected during a short night. A similar influence of the photoperiod was also observed in the case of *TNFα* gene expression in the control animals. A higher level of *TNFα* gene expression during the long night was also observed in the control group of animals [14].

Our study also showed the expression of mRNA encoding proinflammatory cytokines receptors in the AP gland. This expression is different depending on the duration of the inflammatory signal. In our study only an acute inflammation induced the expression of both IL-1 receptors gene, while a prolonged inflammation did not affect this expression, in contrast to IL-6 receptors which are enhanced during an acute as well during prolonged inflammation. This may be due to the fact that *IL-1β* gene expression in AP is enhanced only in the acute inflammation, while *IL-6* may be a part of an acute and prolonged inflammation. Despite the inflammation induced TNF expression during both type of inflammation, its receptors gene expression is enhanced only during the acute immune stress. It can be an effect of TNF specificity. As it was mentioned, this cytokine is significantly elevated during an autoimmunological disease [33,41], so the constant level of receptor gene expression during prolonged inflammation may be to protect the cell from overstimulation.

It is well documented that an acute as well prolonged LPS administration influence gonadotropins secretion in sheep [6,10]. In the present study we demonstrated that inflammatory dependent changes in the gene expression of proinflammatory cytokines and their receptors are accompanied by the changes in the secretory activity of ovine AP. The reduction of LH secretion under an acute and prolonged immune stress was found. On the other hand, the FSH secretion was raised after a repeated LPS injection. It was previously reported that cytokines acting in an autocrine and paracrine ways in the pituitary, affect the hormone secretion and pituitary growth. IL-1β, in dose-dependent manner, inhibits the growth of rat pituitary cells and this effect was reversed by IL-1 receptor agonist. Moreover, IL-1β stimulated most rat pituitary hormones secretion [40]. On the other hand, the picomolar concentration of IL-6 may enhance prolactin release, growth hormone (GH) and LH from dispersed AP cells [42]. IL-6 stimulates GH release from rat hemipituitaries [43] and GH and prolactin release from lactosomatotrophic cells [44]. It has been stated that TNF blunts the release of adrenocorticotropic hormone (ACTH) and other pituitary hormones in response to hypothalamic factors in AP cells culture. TNF treatment of hemipituitaries increased ACTH, GH and thyroid-stimulating hormone (TSH) secretion, while prolactin secretion was not affected. However, those results were dose-dependent [40].

It is worth mentioning that the reduction of LH secretion during an acute and prolonged inflammation could result from the suppression of GnRH secretion in the hypothalamus. Our previous study showed that both the acute [45,46] and prolonged [47] inflammation reduced GnRH secretion in the hypothalamus of ewes. Moreover, the suppression of LH secretion during the acute inflammation could result from a reduced expression of *GnRHR* in the AP. The amount of GnRHR determining the ability and storage strength of the pituitary gonadotropes in response to GnRH [48]. It was previously found that during inflammation the pituitary sensitivity to GnRH stimulation [9] and the expression of *GnRHR* in ewes were suppressed [49,50]. It is believed that the lower *GnRHR* gene expression in the pituitary during an immune/inflammatory challenge is mainly due to the lower secretion of the hypothalamic GnRH [1,45,46,47], which is one of the most important regulator of its own receptor expression.

Although inflammation interrupts GnRH/LH regulation, it has been confirmed that LPS injection may elevate the FSH concentration [51]. Our results show that prolonged inflammation caused a higher blood concentration of FSH, which is similar with our previous study in which the stimulatory effect of LPS was observed from the second injection [10].

## 4. Materials and Methods

### 4.1. Animals and Experimental Design

The experiments were carried out on Blackhead ewes (n = 24) during long day (LD) (8:16, June). The animals were maintained indoors in individual pens and were exposed to natural daylight present at 52° N latitude and 21° E longitude. The ewes were maintained in good conditions, i.e., their body condition was estimated at 3 in a five-point scale [52] and the animals were acclimated to the experimental conditions for one month. The ewes had constant visual contact with each other in order to avoid isolation stress. The animals were fed a constant diet of commercial concentrates with hay and water available ad libitum, according to the recommendations proposed by the National Research Institute of Animal Production for adult ewes [53].

**Figure 6:**
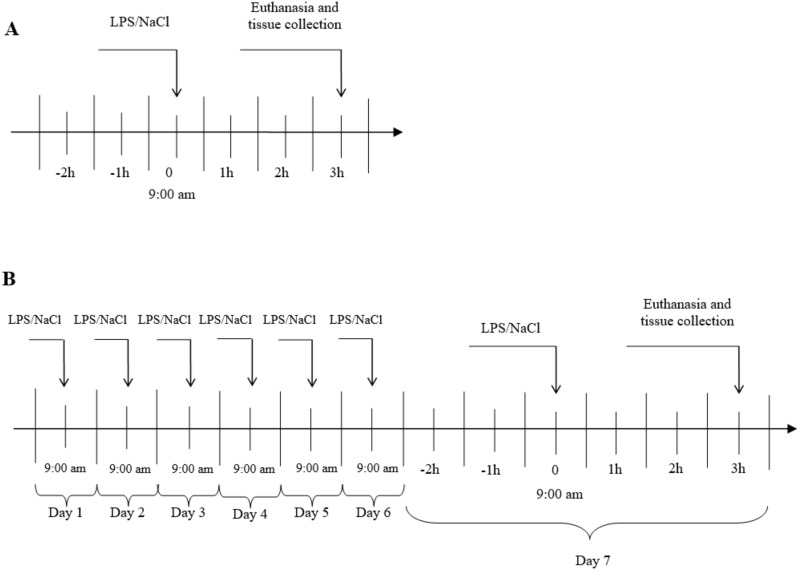
General procedure of experiment. **A**- Acute inflammation, **B**- prolonged inflammation, LPS- lipopolisaccaride S from E. coli 055:B5 in 400 ng•kg^−1^ dose.

The experiments were carried out on 3 years old Blackhead ewes (n = 24) from the same herd, during long day (LD) (8:16, June). During the first experiment, an acute stress was induced by single injection into the jugular vein, an appropriate volume of LPS from E. coli 055:B5 (400 ng•kg^-1^) (Sigma-Aldrich, St Louis, MO, USA) dissolved in saline (0.9% *w/v* NaCl) (Baxter, Deerfield, IL, USA). In the second group, a prolonged inflammation was induced by a sevenfold i.v. injection of LPS (one injection per day, at the same hour). The maximum volume of an injected LPS solution (10 mg•L-1) has never exceeded 2.5 mL. The control group received the same volume of NaCl (based on their body weight). The treatments was always administered at 9:00 a.m. The scheme of the conducted experiment is shown in Figure 6. The efficiency of the LPS treatment to induce an inflammatory response in the animal was estimated based on the measurement of the body temperature an hour after the treatment. Jugular blood samples were taken for measurement of LH 15-min before the euthanasia by decapitation which took place three hours after the LPS or saline injection. The brain was immediately removed from the skull, and the AP was dissected, immediately frozen in liquid nitrogen and stored at −80 °C until further assay.

All procedures on animals were performed with the consent of the 3rd Local Ethical Commission of Warsaw University of Life Sciences—SGGW (Warsaw, Poland; authorization no. 43/2011 and 56/2013).

### 4.2. Determination of the Relative Gene Expression

Total RNA from the collected AP was isolated using the NucleoSpin^®^ RNA kit (MACHEREY-NAGEL GmbH and Co, Düren, Germany). Half a tissue of AP was homogenized using a TissueLyser (Qiagen, Hilden, Germany) and then all isolation steps were conducted in accordance with the manufacturer’s instruction. The purity and concentration of the isolated RNA were quantified spectrophotometrically with the use of NanoDrop 1000 instrument (Thermo Fisher Scientific, Waltham, MA, USA). The integrity of the isolated RNA was confirmed by electrophoresis with the use of 1% agarose gel stained with ethidium bromide. The Maxima™ First Strand cDNA Synthesis Kit for RT-qPCR (Thermo Fisher Scientific) was used to perform cDNA synthesis. As a starting material for cDNA synthesis 2 µg of total RNA were used. Real-Time RT-PCR was carried out with the use of the HOT FIREPol EvaGreen^®^ qPCR Mix Plus (Solis BioDyne, Tartu, Estonia) and HPLC-grade oligonucleotide primers (Genomed, Warsaw, Poland) (Table 1). The reactions were run on Rotor-Gene q MDx instrument (Qiagen, Hilden, Germany). The specificity of the amplification was confirmed by a final melting curve analysis. The relative gene expression was calculated using the comparative quantification option (Rasmussen, 2001) of the Rotor-Gene q Series software 1.7. (Qiagen). To obtain the normalized results, the expression of the examined genes was calculated in relation to the average value of three reference genes expression: actin beta (*ACTB)*, glyceraldehyde 3-phosphate dehydrogenase (*GAPDH*), peptidylprolyl isomerase C (*PPIC*). The mean expression of these three housekeeping genes was used to normalize the expression of the analysed genes. The results are presented in arbitrary units, as the ratio of the target gene expression to the mean expression of the housekeeping genes.

## 5. Conclusions

In conclusion, this study showed that an acute and prolonged inflammation differently influences the gene expression of pro-inflammatory cytokines and their receptors in the AP of ewes. Our results suggest that during an acute inflammation the secretory activity of the pituitary may be influenced by locally synthesized IL-1β, IL-6 and TNF-α, whereas during a prolonged inflammation only the local transcription of TNFα is increased in this gland. In parallel with the changes in cytokines gene expression, we observed an influence in the acute and prolonged LPS treatment on gonadotropins secretion. Both the acute and prolonged inflammation, inhibited LH secretion, while only the prolonged LPS treatment influenced FSH secretion. Our results suggest that there are important differences in the way how an acute and prolonged inflammation influence proinflammatory cytokines and their receptors gene expression in the AP of anestrous ewes, which could be reflected by differences in AP secretory activity during these states. Due to the potential of proinflammatory cytokines to disturbing the secretory activity of the pituitary gland, the therapies targeted on the suppression on their local synthesis in the pituitary could have some beneficial effect during the treatment of endocrine disorders which accompany the inflammatory states; however, this requires future detailed studies.

## Figures and Tables

**Table 1:** All genes analyzed by real-time PCR are listed with their full names and abbreviations.

GenBank Acc. No.	Gene	Amplicon Size [bp]	Forward/ Reverse	Sequence 5′→3′	References
NM_001034034	*GAPDH* glyceraldehyde-3-phosphate dehydrogenase	134	forward	AGAAGGCTGGGGCTCACT	[46]
			reverse	GGCATTGCTGACAATCTTGA	
U39357	*ACTB* beta actin	168	forward	CTTCCTTCCTGGGCATGG	[46]
			reverse	GGGCAGTGATCTCTTTCTGC	
NM_001076910	*PPIC* Cyclophilin C	145	forward	TGGCACTGGTGGTATAAGCA	[46]
			reverse	GGGCTTGGTCAAGGTGATAA	
X54796.1	*IL1B* interleukin 1 beta	137	forward	CAGCCGTGCAGTCAGTAAAA	[46]
			reverse	GAAGCTCATGCAGAACACCA	
NM_001206735.1	*IL1R1* Interleukin 1 receptor, type I	124	forward	GGGAAGGGTCCACCTGTAAC	[46]
			reverse	ACAATGCTTTCCCCAACGTA	
NM_001046210.1	*IL1R2* interleukin 1 receptor, type II	161	forward	CGCCAGGCATACTCAGAAA	[46]
			reverse	GAGAACGTGGCAGCTTCTTT	
NM_001009392.1	*IL6* Interleukin 6	165	forward	GTTCAATCAGGCGATTTGCT	[46]
			reverse	CCTGCGATCTTTTCCTTCAG	
NM_001110785	*IL6R* Interleukin 6 receptor	149	forward	TCAGCGACTCCGGAAACTAT	[46]
			reverse	CCGAGGACTCCACTCACAAT	
XM_004016974	*IL6ST* glycoprotein 130	139	forward	GGCTTGCCTCCTGAAAAACC	[14]
			reverse	ACTTCTCTGTTGCCCACTCAG	
NM_001024860	*TNF* Tumor necrosis factor	153	forward	CAAATAACAAGCCGGTAGCC	[46]
			reverse	AGATGAGGTAAAGCCCGTCA	
NM_174674	*TNFRSF1A* Tumor necrosis factor receptor, type 1	137	forward	AGGTGCCGGGATGAAATGTT	[46]
			reverse	CAGAGGCTGCAGTTCAGACA	
NM_001040490	*TNFRSF1B* Tumor necrosis factor receptor, type 2	122	forward	ACCTTCTTCCTCCTCCCAAA	[46]
			reverse	AGAAGCAGACCCAATGCTGT	
NM_001009397	*GnRHR* Gonadotropin releasing hormone receptor	150	forward	TCTTTGCTGGACCACAGTTAT	[47]
			reverse	GGCAGCTGAAGGTGAAAAAG	
X52488	*LHβ* Luteinizing hormone	184	forward	AGATGCTCCAGGGACTGCT	[47]
			reverse	TGCTTCATGCTGAGGCAGTA	
X15493	*FSHβ* Follicle-stimulating hormone	131	forward	TATTGCTACACCCGGGACTT	[47]
			reverse	TACAGGGAGTCTGCATGGTG	

## Abbreviations

ACTH	Adrenocorticotropic hormone
AP	Anterior pituitary
FS	Folliculo-stellate cells
FSH	Follicle-stimulating hormone
GH	Growth hormone
GnRH	Gonadotropin-releasing hormone
IL	Interleukin
LH	Luteinizing hormone
LPS	Lipopolisaccaride S
TNF	Tumor necrosis factor
TSH	Thyroid-stimulating hormone
PT	Pars Tuberalis
